# Epidemiological links between tuberculosis cases identified twice as efficiently by whole genome sequencing than conventional molecular typing: A population-based study

**DOI:** 10.1371/journal.pone.0195413

**Published:** 2018-04-04

**Authors:** Rana Jajou, Albert de Neeling, Rianne van Hunen, Gerard de Vries, Henrieke Schimmel, Arnout Mulder, Richard Anthony, Wim van der Hoek, Dick van Soolingen

**Affiliations:** 1 National Institute for Public Health and the Environment (RIVM), Bilthoven, The Netherlands; 2 KNCV Tuberculosis Foundation, The Hague, The Netherlands; 3 Radboud University Medical Centre, Department of Medical Microbiology, Nijmegen, The Netherlands; Michigan State University College of Veterinary Medicine, UNITED STATES

## Abstract

**Background:**

Patients with *Mycobacterium tuberculosis* isolates sharing identical DNA fingerprint patterns can be epidemiologically linked. However, municipal health services in the Netherlands are able to confirm an epidemiological link in only around 23% of the patients with isolates clustered by the conventional variable number of tandem repeat (VNTR) genotyping. This research aims to investigate whether whole genome sequencing (WGS) is a more reliable predictor of epidemiological links between tuberculosis patients than VNTR genotyping.

**Methods:**

VNTR genotyping and WGS were performed in parallel on all *Mycobacterium tuberculosis* complex isolates received at the Netherlands National Institute for Public Health and the Environment in 2016. Isolates were clustered by VNTR when they shared identical 24-loci VNTR patterns; isolates were assigned to a WGS cluster when the pair-wise genetic distance was ≤ 12 single nucleotide polymorphisms (SNPs). Cluster investigation was performed by municipal health services on all isolates clustered by VNTR in 2016. The proportion of epidemiological links identified among patients clustered by either method was calculated.

**Results:**

In total, 535 isolates were genotyped, of which 25% (134/535) were clustered by VNTR and 14% (76/535) by WGS; the concordance between both typing methods was 86%. The proportion of epidemiological links among WGS clustered cases (57%) was twice as common than among VNTR clustered cases (31%).

**Conclusion:**

When WGS was applied, the number of clustered isolates was halved, while all epidemiologically linked cases remained clustered. WGS is therefore a more reliable tool to predict epidemiological links between tuberculosis cases than VNTR genotyping and will allow more efficient transmission tracing, as epidemiological investigations based on false clustering can be avoided.

## Introduction

The Netherlands is a low incidence country with 5.2 tuberculosis cases per 100,000 inhabitants. In 2016, 889 tuberculosis patients were notified, which was an increase of 3% compared to 2015 due to the high influx of asylum seekers from high tuberculosis incidence countries, i.e. Eritrea, Ethiopia, and Somalia [1]. In line with the End TB strategy [2] and the framework towards tuberculosis elimination in low-incidence countries [3], the Netherlands developed a National Tuberculosis Control Plan aiming to reduce tuberculosis incidence and transmission with 25% both in five years [4]. Whole genome sequencing (WGS) was particularly identified as a promising tool to better measure transmission and control tuberculosis [5].

All *M*. *tuberculosis* complex isolates in the Netherlands are sent to the National Tuberculosis Reference Laboratory for genotyping. Variable number of tandem repeat (VNTR) genotyping is the current DNA fingerprinting method and has been routinely applied in the Netherlands since 2009 [6, 7]. Isolates are considered clustered when they share identical 24-loci VNTR patterns. The information on clustering of cases is reported on a weekly basis to municipal health services to guide epidemiological investigations. Epidemiological links between patients suggested by VNTR typing are investigated by tuberculosis public health nurses from the municipal health services, with the aim of identifying transmission and preventing further spread of the disease. In 2015, only 23% of cases clustered by VNTR genotyping could be epidemiologically linked by municipal health services [8]. This low degree of confirmation is assumed to be partly due to false clustering as a result of the low rate of change of VNTR loci in the genome of *M*. *tuberculosis*, rather than insufficient epidemiological investigations. This might be especially true for isolates from patients originating from high-prevalence geographic areas such as the Horn of Africa, where transmission is less efficiently interrupted by tuberculosis control efforts and strains may be genetically highly conserved [9, 10].

WGS of *M*. *tuberculosis* isolates potentially has a higher resolution than VNTR genotyping as a much larger fraction of the genome, more than four mega base pairs of DNA, is analysed for diversity [11, 12]. However, it is unclear whether the general genetic turnover in *M*. *tuberculosis* is rapid enough to study the transmission of tuberculosis efficiently. In 2016, a four-year nationwide WGS project was initiated in the Netherlands, which has been to date applied for research purposes only. In this project, WGS runs simultaneously with the conventional VNTR genotyping, while cluster investigation by municipal health services remains to be performed based on VNTR genotyping, until WGS is implemented in the Netherlands. In this study, we aim to 1) compare clustering on basis of VNTR versus WGS, and 2) compare the degree of confirmed epidemiological links by municipal health services on basis of information from both typing methods.

## Materials and methods

### Study population

All *M*. *tuberculosis* complex isolates cultured in the Netherlands between January 1, 2016 and December 31, 2016, were included in this study. VNTR genotyping and WGS were simultaneously performed for all included samples. Laboratory cross contaminations (i.e. isolates with an identical VNTR pattern received within one week from the same peripheral laboratory), *M*. *bovis* Bacillus Calmette-Guérin (BCG), as well as multiple isolates from the same patient were excluded.

### Molecular typing

DNA used for both typing methods was isolated from a positive Mycobacteria Growth Indicator Tube and purified with the QIAamp DNA mini kit method (QIAGEN GmbH, Hilden, Germany) under BSL-3 laboratory conditions. VNTR genotyping was performed as described earlier [6, 7] and VNTR data were collected from BioNumerics version 7.6.2. Isolates sharing identical 24-loci VNTR patterns were assigned to the same VNTR cluster.

In parallel with VNTR genotyping, *M*. *tuberculosis* complex DNA samples were also sequenced on an Illumina HiSeq2500 sequencer that generated paired-end reads of 125-bp. A minimum sample yield of 350 Mb was required to achieve an average sequencing coverage of 80 reads for *M*. *tuberculosis* samples considering the 4.4 Mb genome size. Reads were mapped unpaired against the H37Rv reference genome version 3.0 (GenBank accession number AL123456.3) using Bowtie2 in Breseq version 0.28.1 [13]. Single nucleotide polymorphisms (SNPs) were detected with Breseq using standard settings, i.e. a minimum allele frequency of 80% and a minimum coverage of five reads. Bam files of all sequenced samples are available in the European Nucleotide Archive (ENA) under accession number PRJEB25592.

### Data collection and analysis

Tuberculosis is a notifiable disease and cases are reported to the Netherlands Tuberculosis Register. Data on patient characteristics, i.e. pulmonary tuberculosis (PTB)/ extra pulmonary tuberculosis (ETB), geographical region of residence in the Netherlands, age, gender, ethnicity, rural/urban living, risk group, resistance, and co-morbidities were collected from this register. The register also includes data on epidemiological links as established by municipal health services by interviews with tuberculosis patients. However, since these data are entered into the Netherlands Tuberculosis Register at a relatively late stage, municipal health services were in this study actively contacted by phone to obtain results of cluster investigations on patients that had an isolate with an identical VNTR pattern with another patient in 2016. Public health nurses in the Netherlands perform VNTR cluster investigations according to a standardized questionnaire. In this study, only confirmed epidemiological links were included, which is in the Netherlands Tuberculosis Register defined as A) patients know each other by name and were present on the same time and place, or B) patients do not know each other by name, but the patients within the same VNTR cluster were present in the same period on the same address/location (e.g. school, work, gym, café).

R statistics version 3.3.2 [14] was applied for WGS data analysis, excluding genetic regions annotated as PE/PPE, PGRS, pks, esx, repeat, polyketide, or transposase in the gene name and/or gene product description in the annotated Genome Difference files produced by Breseq. Isolates with a maximum pair-wise distance of 12 SNPs in the proportion of the genome analysed, were assigned to a WGS cluster, as suggested earlier by Walker et al., 2013 [12]. We investigated whether isolates from VNTR clustered patients with confirmed epidemiological links had a pair-wise genetic distance of ≤ 12 SNPs when WGS was applied. The number of (transmission) events within a VNTR cluster was calculated by the number of patients within the respective VNTR cluster minus the index patient (n-1 method). Fastq.gz files were uploaded to PhyResSe [15] to assign a lineage to each isolate. The Chi-square test was used to analyse differences between patients clustered by WGS and patients that were not; Fisher’s exact test was used for cell counts below five.

## Results

### Patient and strain characteristics

In 2016, 535 *M*. *tuberculosis* complex isolates were subjected to both WGS and VNTR genotyping; data from the Netherlands Tuberculosis Register was missing for eight patients. The median age of patients was 35 years (range 0–102) and 60% were men. The majority of patients were first generation migrants (78.4%) and more than half (62.3%) of the patients had PTB or the combination with ETB (Table 1).

**Table 1:** Patient characteristics of 527/535 patients with complete data from the Netherlands Tuberculosis Register.

	Study population (n = 527)	WGS clustered		P-value
		Clustered (n = 76)	Non-clustered (n = 451)	
**Age in years, median (range)**	35 (0–102)	23 (0–89)	37 (13–102)	-
**Age in categories (in years)** 0–24 25–44 45–64 65+	127 (23.7%) 215 (40.8%) 105 (19.9%) 80 (15.2%)	42 (55.3%) 23 (30.3%) 7 (9.2%) 4 (5.3%)	85 (18.8%) 192 (42.6%) 98 (21.7%) 76 (16.9%)	< 0.001 0.062 0.016 0.013
**Gender, male**	316 (60%)	55 (72.4%)	261 (57.9%)	0.018
**Rural living**	373 (70.8%)	59 (77.6%)	314 (69.6%)	0.162
**Diagnosis** PTB ETB PTB+ETB	255 (48.4%) 200 (38%) 72 (13.7%)	41 (53.9%) 24 (31.6%) 11 (14.5%)	214 (47.5%) 176 (39%) 61 (13.5%)	0.292 0.192 0.756
**Resistance** Isoniazid mono-resistance Rifampicin mono-resistance Pyrazinamide mono-resistance Multidrug-resistant	30 (5.7%) 2 (0.4%) 15 (2.8%) 12 (2.3%)	2 (2.6%) 0 (0%) 0 (0%) 0 (0%)	28 (6.2%) 2 (0.4%) 15 (3.3%) 12 (2.7%)	0.217 0.397 0.127 0.166
**Ethnicity** Dutch First generation migrant ^a^ Second generation migrant ^b^ Unknown	65 (12.3%) 413 (78.4%) 36 (6.8%) 13 (2.5%)	6 (7.9%) 64 (84.2%) 6 (7.9%) 0 (0%)	59 (13.1%) 349 (77.4%) 30 (6.7%) 13 (2.9%)	0.229 0.216 0.646 -
**Risk group** Contact of tuberculosis patient Immigrant ^c^ Asylum seeker ^d^ Undocumented migrant Homeless Alcohol addict Drug addict Prisoner Travel to endemic regions > 3 mo	41 (7.8%) 35 (6.6%) 106 (20.1%) 21 (4%) 14 (2.7%) 4 (0.8%) 6 (1.1%) 11 (2.1%) 15 (2.8%)	23 (30.3%) 3 (3.9%) 37 (48.7%) 6 (7.9%) 2 (2.6%) 1 (1.3%) 0 (0%) 0 (0%) 0 (0%)	18 (4%) 32 (7.1%) 69 (15.3%) 15 (3.3%) 12 (2.7%) 3 (0.7%) 6 (1.3%) 11 (2.4%) 15 (3.3%)	< 0.001 0.280 < 0.001 0.076 0.471 0.390 0.281 0.181 0.127
**Comorbidity** Diabetes Malignancy Renal failure Organ transplantation	28 (5.3%) 19 (3.6%) 8 (1.5%) 2 (0.4%)	3 (3.9%) 1 (1.3%) 0 (0%) 0 (0%)	25 (5.5%) 18 (4%) 8 (1.8%) 2 (0.4%)	0.783 0.500 0.609 > 0.999
**Lineages** ^**e**^ EAI Beijing Delhi/CAS EAS LAM Cameroon Haarlem S-type TUR Uganda Ural West African 1 West African 2 No lineage assigned by PhyResSe	60 (11.2%) 43 (8.3%) 117 (22.5%) 127 (24.4%) 57 (11%) 4 (0.8%) 78 (15%) 12 (2.3%) 2 (0.4%) 3 (0.6%) 7 (1.3%) 1 (0.2%) 2 (0.4%) 7 (1.3%)	3 (3.9%) 0 (0%) 31 (40.8%) 16 (21.1%) 5 (6.6%) 0 (0%) 17 (22.4%) 4 (5.3%) 0 (0%) 0 (0%) 0 (0%) 0 (0%) 0 (0%) 0 (0%)	57 (12.8%) 43 (9.7%) 86 (19.4%) 111 (25%) 52 (11.7%) 4 (0.9%) 61 (13.7%) 8 (1.8%) 2 (0.5%) 3 (0.7%) 7 (1.6%) 1 (0.2%) 2 (0.5%) 7 (1.6%)	0.030 0.001 < 0.001 0.208 0.198 > 0.999 0.044 0.080 > 0.999 > 0.999 0.601 > 0.999 > 0.999 -

Chi-square test was used to generate p-values; Fisher’s exact test was used for cell counts below five.

PTB: pulmonary tuberculosis; ETB: extra-pulmonary tuberculosis; EAI: East-African-Indian; CAS: Central-Asian; EAS: Euro-American; LAM: Latin American-Mediterranean

^a^ This is in the Netherlands Tuberculosis Register defined as a person was foreign-born and at least one parent was foreign-born.

^b^ This is in the Netherlands Tuberculosis Register defined as a person born in the Netherlands, of whom at least one parent was foreign-born.

^c^ This is in the Netherlands Tuberculosis Register defined as a person with a legal residence status other than a tourist or refugee/asylum seeker, who is subject to the immigrant screening regulations and who resides in the Netherlands less than 2.5 years.

^d^ This is in the Netherlands Tuberculosis Register defined as a person who is subject to regulations relating to the screening of asylum seekers, already has a valid residence status as an asylum seeker or is still in the asylum seeker procedure and has been residing in the Netherlands less than 2.5 years.

^e^ These data are (RIVM) laboratory data and were available for all 535 isolates of which 520 isolates belong to *M*. *tuberculosis*; 76 were WGS clustered and 444 were not.

Of the 535 isolates, 97.2% (520/535) were *M*. *tuberculosis*, 2.4% (13/535) *M*. *bovis*, 0.2% (1/535) *M*. *caprae*, and 0.2% (1/535) *M*. *orygis*. Twenty-four percent (127/520) of *M*. *tuberculosis* isolates belonged to the EAS lineage, followed by 22.5% belonging to the Delhi/CAS lineage (Table 1). Ten isolates had a relatively low mean coverage below 20 reads; the mean coverage of the remaining 525 isolates was 117 (range 22–340).

The median age of the WGS clustered cases was 23 years (range 0–89) compared to 37 years (range 13–102) of non-WGS clustered cases. WGS clustered cases were more likely to be male (72.4% vs 57.9%, p = 0.018), contact of a tuberculosis patient (30.3% vs 4%, p<0.001), and asylum seekers (48.7% vs 15.3%, p<0.001) with associated Delhi/CAS lineage (40.8% vs 19.4%, p<0.001). The Haarlem (22.4% vs 13.7%, p = 0.044) and S-type (5.3% vs 1.8%, p = 0.080) lineages were also more frequently observed among isolates of the WGS clustered cases (Table 1).

### VNTR versus WGS clustering

Using VNTR genotyping, 46% (246/535) had a unique pattern, 29% (155/535) were clustered with isolates from before 2016, and 25% (134/535) were clustered with another isolate in 2016. The 134 isolates clustering with another isolate in 2016 belonged to 41 different VNTR clusters with cluster sizes ranging from 2–21 isolates; 25/41 clusters consisted of two isolates.

**Fig 1:**
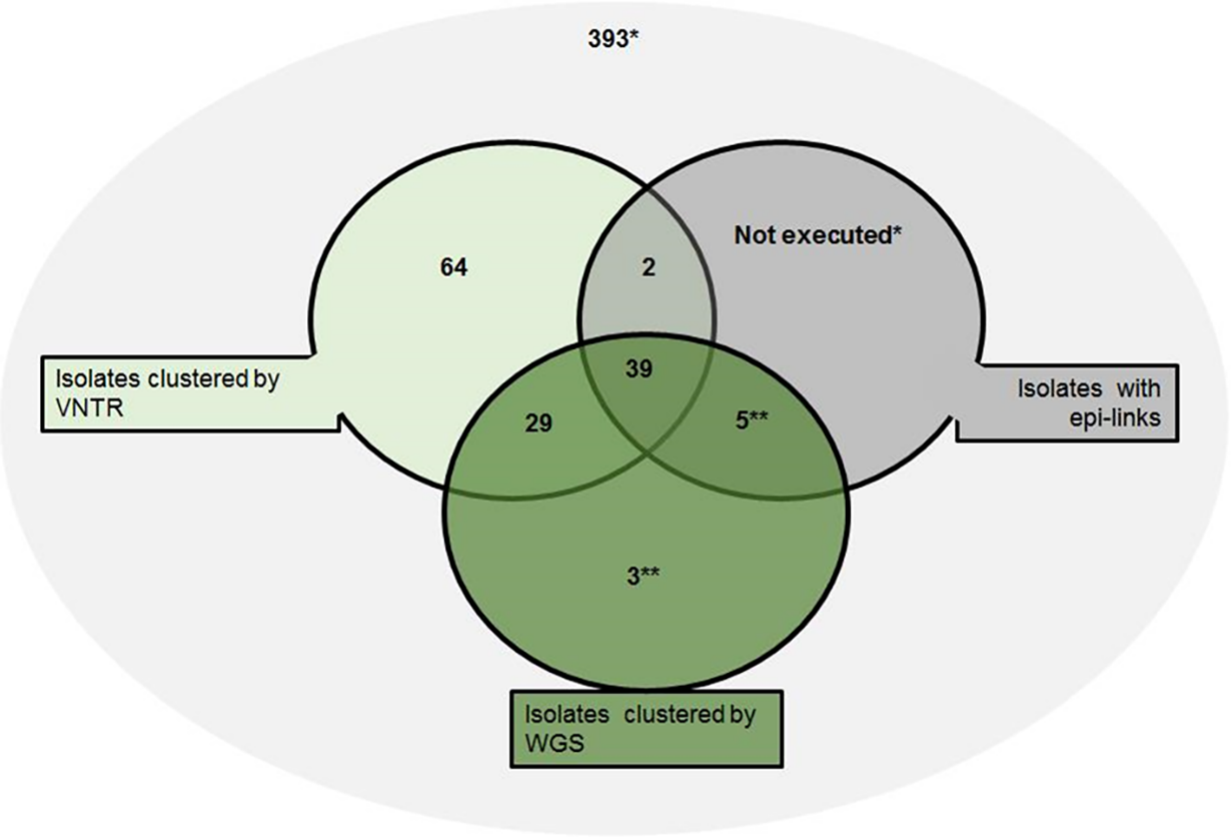
Venn diagram of VNTR and WGS typing of 535 *M*. *tuberculosis* complex isolates from the Netherlands and confirmed epidemiological links in cluster investigation. * Isolates with unique VNTR profiles in 2016 were not investigated for epidemiological links. ** Epidemiological link information is based on geographical proximity, as cluster investigation was not conducted for isolates with different VNTR profiles.

WGS clustered 14.2% (76/535) of the isolates. Sixty-eight of these isolates were also clustered by VNTR and an additional three clusters comprising a total of eight cases were clustered by WGS only; two clusters of three cases and one of two cases (Fig 1). The VNTR profiles within each of these WGS clusters were however highly similar; isolates from two clusters varied at one VNTR locus and isolates in the other cluster varied by maximum two VNTR loci. The remaining 393 isolates were not clustered by either method, resulting in 86.2% (393+68/535) concordance between the two DNA fingerprint methods.

### Cluster investigation

**Fig 2:**
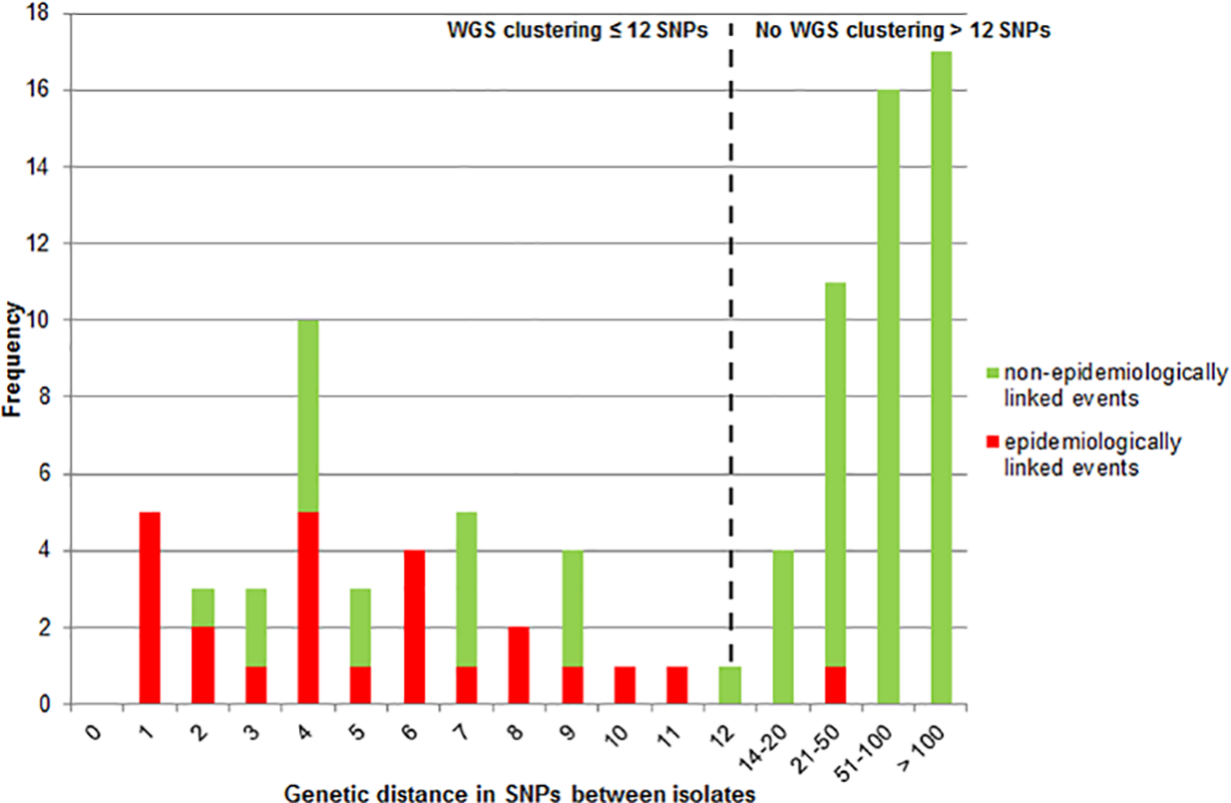
Correlation between genetic distances in SNPs and events for which an epidemiological link was confirmed (red) or not (green) of all 134 VNTR clustered isolates in 2016. The frequencies on the y-axis represent the number of events (n-1) within VNTR clusters rather than the number of isolates. The dashed line indicates the threshold of ≤ 12 SNPs used to rule in transmission in this study.

As described earlier, cluster investigation by municipal health services is currently only performed on VNTR clustered cases, as WGS is not yet routinely implemented in the Netherlands. In 2016, 134 patients were clustered by VNTR; cluster investigation resulted in 41 patients to be epidemiologically linked and for the remaining 93 patients an epidemiological link could not be identified. The proportion of confirmed epidemiological links identified in WGS clustered isolates was 57.4% (39/68) compared to 30.6% (41/134) among VNTR clustered cases. Among the 66 patients that were not clustered by WGS, two were epidemiologically linked, but their isolates showed a genetic distance of 27 SNPs (Figs 1 and 2).

The 41 epidemiologically linked patients represented 25 transmission events that all, except for one, had ≤ 12 SNPs genetic distance (range 1–11 SNPs). The genetic distance for events between patients that could not be epidemiologically linked was up to 201 SNPs; 2–12 SNPs for 29 of the 93 non-epidemiologically linked patients that did cluster by WGS, and 14–201 SNPs for 64 of the 93 non-epidemiologically linked patients that were not clustered by WGS (Fig 2).

The confirmation of epidemiological links was also compared with the information on the geographic spread and infectiousness (PTB or ETB) of the patients. All 41 patients with a confirmed epidemiological link were from the same geographic area and were diagnosed with either PTB or the combination of PTB/ETB. Non-epidemiologically linked cases were in general geographically more distant from each other (S1 and S2 Tables).

Cluster investigation was not performed on the three additional clusters containing in total eight patients identified by WGS only, as they were not clustered by VNTR. Based on the geographic proximity and infectiousness of tuberculosis, for two of the three additional clusters comprising five patients, an epidemiological link appears likely. Thus, with VNTR 134 epidemiological investigations were required to identify 41 epidemiologically linked patients, which is a yield of 30.6%, whereas WGS would have required 76 (68+8) epidemiological investigations to identify 44 (39+5) epidemiologically linked cases, a yield of 57.9% (Fig 1).

## Discussion

This is the first prospective, population-based study to quantify the benefit of WGS over the current standard VNTR genotyping on a nation-wide collection of more than 500 *M*. *tuberculosis* complex isolates. Typing by WGS increased efficiency by reducing the number of cases requiring cluster investigations by half, while the degree of confirmed epidemiologically linked cases doubled. Furthermore, WGS clustered an additional eight cases that were not clustered by VNTR genotyping, five of which based on geographical proximity could possibly belong to chains of transmission.

Previous studies already indicated that VNTR can be misleading and some VNTR clusters can be identified as false when WGS is applied [16–23]. In fact, isolates from one of the VNTR clusters in this study had genetic distance of more than 200 SNPs by WGS, indicating that VNTR occasionally clusters isolates with relatively large genetic distances. Our results are comparable to a previous study performed in Switzerland, a low tuberculosis incidence country like the Netherlands, which showed that around 48% of the cases clustered by VNTR remained clustered by WGS when applying a cut-off of 12 SNPs for clustering [24].

A limitation of our study is that cluster investigation by municipal health services was only included for patients with isolates clustering on basis of VNTR with other isolates in 2016, which can lead to an underestimation of transmission. Of the 93 patients clustered by VNTR for which no epidemiological link could be confirmed, 29 also clustered by WGS. Almost all of these 29 patients were asylum seekers from Eritrea/Ethiopia. A recent study from the Netherlands showed that transmission among asylum seekers from the Horn of Africa most likely occurred during the escape route, but that a proportion of these patients might been infected after arrival in the destination country [10]. This is confirmed by a recent study from Norway suggesting that among 25% of immigrants from high incidence countries recent transmission in the destination country rather than import is likely [25]. In general, cluster investigation also misses a considerable proportion of epidemiological links. It is particularly challenging to perform cluster investigation among immigrants and asylum seekers due to language/cultural barriers and/or frequent migration within the Netherlands, and epidemiological links might have been missed [26]. However, it remains possible that these patients were not epidemiologically linked, at least not within the Netherlands, as previous studies have also observed a genetic distance of ≤12 SNPs between non-linked patients [12, 19, 21, 27]. Even if all 29 cases were incorrectly clustered by WGS or epidemiological links were missed among these patients, using this technique instead of VNTR to initiate cluster investigations would have reduced the number of investigations performed by half.

Furthermore, 15 of the 535 (2.8%) isolates had WGS data originating from subspecies other than *M*. *tuberculosis* and ten *M*. *tuberculosis* isolates had a relatively low mean coverage. Re-analysing the data by excluding these samples did not significantly affect the results (data not shown) as only one of the low coverage isolates was part of the 134 isolates clustering by VNTR in 2016. This low coverage isolate belonged to the epidemiologically linked patient with 27 SNPs genetic distance with its pair, meaning this link was missed by WGS due to bad sequence quality.

The main strength of this study is that all *M*. *tuberculosis* complex isolates in the Netherlands are genotyped at one national tuberculosis reference laboratory, making the results of this study generalizable to the entire patient population. Also, all municipal health services perform extensive source case tracing and contact investigation and support patients on a regular basis. The results of these investigations together with a wide spectrum of patient characteristics are reported to the Netherlands Tuberculosis Register, increasing the validity of this study. However, information bias remains possible due to language/cultural barriers when interviewing patients, which include many immigrants and asylum seekers.

Currently there is no international standard for the SNPs distance cut-off to rule in a possible transmission, and various cut-offs have been applied in studies in different countries, which limits the ability to compare data [18–20, 28–30]. In the study of Walker et al. 2014, a sensitivity analysis was performed using several SNP thresholds, and results showed that a threshold of one SNP increased sensitivity to identify epidemiological links to 59% compared to 42% when applying the 12 SNP threshold [27]. Based on our population-based study, the cut-off of 12 SNPs seems to be valid in a low incidence country like the Netherlands when compared to results of cluster investigations by municipal health services. The different thresholds applied worldwide are however influenced by the WGS pipeline used, which can vary in for example the stringency method (i.e. the genetic regions excluded during WGS analysis), the minimum mean sample coverage accepted, the minimum number of reads that support SNPs, and the minimum allele frequency to call SNPs. International standardization is needed on all these factors to ensure that the SNPs cut-offs applied to WGS clustering are comparable between WGS studies, allowing the efficient investigation of cross border transmission.

In conclusion, the clustering of tuberculosis cases in 2016 was reduced by half on the basis of WGS compared to VNTR, while retaining the number of epidemiological links. This observation confirms that VNTR genotyping leads to a considerable proportion of false clustering, as was already suggested by the low confirmation of epidemiological links identified by municipal health services in the Netherlands. The lower degree of clustering by WGS will prevent unnecessary cluster investigations. Moreover, the much higher degree of confirmed epidemiological links within WGS clusters will contribute to improved understanding of tuberculosis transmission. Therefore, basing epidemiological investigations on WGS typing can support tuberculosis elimination in a low incidence country such as the Netherlands.

## Supporting information

S1 Table Characteristics of the 41 epidemiologically linked patients presented per VNTR cluster. (DOCX)

S2 Table Characteristics of the 93 non-epidemiologically linked patients, presented per VNTR cluster. (DOCX)
