# Correction: Epidemiological links between tuberculosis cases identified twice as efficiently by whole genome sequencing than conventional molecular typing: A population-based study

**DOI:** 10.1371/journal.pone.0197556

**Published:** 2018-05-14

**Authors:** Rana Jajou, Albert de Neeling, Rianne van Hunen, Gerard de Vries, Henrieke Schimmel, Arnout Mulder, Richard Anthony, Wim van der Hoek, Dick van Soolingen

There is an error in the listed author names in the citation. The correct citation is: Jajou R, de Neeling A, van Hunen R, de Vries G, Schimmel H, Mulder A, et al. (2018) Epidemiological links between tuberculosis cases identified twice as efficiently by whole genome sequencing than conventional molecular typing: A population-based study. PLoS ONE 13(4): e0195413. https://doi.org/10.1371/journal.pone.0195413
