# Supplementary material for: Epidemiological links between tuberculosis cases identified twice as efficiently by whole genome sequencing than conventional molecular typing: A population-based study
Source: PLoS One. 2018 Apr 4;13(4):e0195413. doi: 10.1371/journal.pone.0195413 (PMC5884559; doi:10.1371/journal.pone.0195413)
Supplement: S1 Table — (DOCX) [file pone.0195413.s001.docx]

**S1 Table Characteristics of the 41 epidemiologically linked patients presented per VNTR cluster**

|  | **WGS clustered (n=39)** | | | | | | | | | | | | | | | **Non-WGS clustered (n=2)** |
| --- | --- | --- | --- | --- | --- | --- | --- | --- | --- | --- | --- | --- | --- | --- | --- | --- |
| **VNTR cluster ID** | **A** | **B** | **C** | **D** | **E** | **F** | **G** | **H** | **I** | **J** | **K** | **L** | **M** | **N** | **O** | **Q** |
| **Cluster size** | 2 | 2 | 2 | 3 | 3 | 4 | 4 | 2 | 3 | 2 | 3 | 3 | 2 | 2 | 2 | 2 |
| **Age in years**  0-14  15-24  25-34  35-44  45-54  55-64  65+ | -  -  -  -  2  -  - | -  2  -  -  -  -  - | -  1  -  1  -  -  - | -  3  -  -  -  -  - | -  2  1  -  -  -  - | -  3  -  1  -  -  - | -  2  -  -  1  -  1 | -  -  -  2  -  -  - | -  3  -  -  -  -  - | 1  1  -  -  -  -  - | -  -  2  1  -  -  - | 1  -  1  -  -  1  - | 2  -  -  -  -  -  - | -  2  -  -  -  -  - | -  1  1  -  -  -  - | -  -  -  2  -  -  - |
| **Gender**  Male  Female | 2  - | 2  - | 1  1 | 3  - | 2  1 | 4  - | 2  2 | 1  1 | 2  1 | 1  1 | 3  - | 2  1 | 1  1 | 2  - | 1  1 | 2  - |
| **Geographic region**  Groningen, Friesland, Drenthe  Overijssel, Gelderland  Utrecht  North-Holland, Flevoland  South-Holland  South  South East | -  -  2  -  -  -  - | -  2  -  -  -  -  - | -  -  -  -  2  -  - | -  -  -  -  3  -  - | -  -  -  3  -  -  - | -  4  -  -  -  -  - | -  -  -  -  -  4  - | -  -  -  2  -  -  - | -  -  -  -  -  -  3 | -  -  -  -  2  -  - | -  -  -  3  -  -  - | -  -  -  -  3  -  - | -  -  -  -  2  -  - | 1  1  -  -  -  -  - | -  -  -  -  2  -  - | -  -  -  2  -  -  - |
| **Ethnicity**  Dutch  First generation migrant  Second generation migrant | -  2  - | 2  -  - | -  -  2 | -  3  - | -  3  - | -  4  - | -  4  - | -  2  - | -  3  - | -  -  2 | -  3  - | -  2  1 | -  2  - | -  2  - | -  2  - | -  2  - |
| **Country of birth**  Netherlands  Surinam  Eritrea/Ethiopia  Somalia  Indonesia  Ghana  Libya  Morocco  Pakistan | -  2  -  -  -  -  -  -  - | 2  -  -  -  -  -  -  -  - | 2  -  -  -  -  -  -  -  - | -  -  3  -  -  -  -  -  - | -  -  3  -  -  -  -  -  - | -  -  4  -  -  -  -  -  - | -  -  -  4  -  -  -  -  - | -  -  -  -  2  -  -  -  - | -  -  -  3  -  -  -  -  - | 2  -  -  -  -  -  -  -  - | -  -  -  -  -  2  1  -  - | 1  -  -  -  -  -  -  2  - | -  -  -  2  -  -  -  -  - | -  -  2  -  -  -  -  -  - | -  -  -  -  -  -  -  -  2 | -  2  -  -  -  -  -  -  - |
| **Risk group**  Contact of tuberculosis patient  Immigrant  Asylum seeker  Undocumented migrant  Homeless  Alcohol addict | 1  -  -  -  -  1 | 1  -  -  -  -  - | 2  -  -  -  -  - | -  -  3  -  -  - | 2  -  2  -  -  - | 3  -  4  -  -  - | 3  -  -  -  -  - | -  1  -  1  -  - | 2  -  3  -  -  - | 1  -  -  -  -  - | -  -  -  3  1  - | 2  -  -  -  -  - | 1  -  1  -  -  - | -  -  2  -  -  - | 1  -  -  2  -  - | 1  -  -  -  1  - |
| **Diagnosis**  PTB  ETB  PTB+ETB | 2  -  - | -  1  1 | 2  -  - | 1  2  - | 3  -  - | 4  -  - | 3  1  - | 1  -  1 | 2  -  1 | 1  -  1 | -  2  1 | 3  -  - | 1  -  1 | 1  1  - | 1  1  - | 2  -  - |

PTB: pulmonary tuberculosis; ETB: extra-pulmonary tuberculosis
