# Supplementary material for: Epidemiological links between tuberculosis cases identified twice as efficiently by whole genome sequencing than conventional molecular typing: A population-based study
Source: PLoS One. 2018 Apr 4;13(4):e0195413. doi: 10.1371/journal.pone.0195413 (PMC5884559; doi:10.1371/journal.pone.0195413)
Supplement: S2 Table — (DOCX) [file pone.0195413.s002.docx]

**S2 Table Characteristics of the 93 non-epidemiologically linked patients, presented per VNTR cluster**

|  | WGS clustered (n=29) | | | | | | | | | | Non-WGS clustered (n=64) | | | | | | | | | | | | | | | | | | | | | |
| --- | --- | --- | --- | --- | --- | --- | --- | --- | --- | --- | --- | --- | --- | --- | --- | --- | --- | --- | --- | --- | --- | --- | --- | --- | --- | --- | --- | --- | --- | --- | --- | --- |
| VNTR cluster ID | **1** | **2** | **3** | **4** | **5** | **6** | **7** | **8** | **9** | **10** | **a** | **b** | **c** | **d** | **e^1^** | **f** | **g^2^** | **h^3^** | **i** | **j** | **k** | **l** | **m** | **n** | **o** | **p** | **q** | **r** | **s^4^** | **t** | **u** | **v** |
| Cluster size | 6 | 4 | 4 | 2 | 2 | 3 | 2 | 2 | 2 | 2 | 2 | 3 | 2 | 4 | 3 | 3 | 21 | 14 | 2 | 3 | 2 | 3 | 4 | 2 | 3 | 2 | 2 | 2 | 4 | 2 | 2 | 2 |
| Age in years  0-14  15-24  25-34  35-44  45-54  55-64  65+ | -  -  2  3  -  -  1 | -  1  2  1  -  -  - | -  2  -  2  -  -  - | -  1  -  -  -  -  1 | 1  -  1  -  -  -  - | -  3  -  -  -  -  - | -  2  -  -  -  -  - | -  -  -  -  -  1  1 | 1  1  -  -  -  -  - | -  2  -  -  -  -  - | -  2  -  -  -  -  - | -  -  1  -  2  -  - | -  -  -  -  2  -  - | -  1  -  3  -  -  - | -  2  1  -  -  -  - | -  -  1  -  -  1  1 | 1  13  4  2  -  1  - | -  6  2  3  2  -  1 | -  -  -  -  -  1  1 | -  1  1  1  -  -  - | -  1  1  -  -  -  - | -  1  1  -  1  -  - | -  -  -  -  -  -  4 | -  2  -  -  -  -  - | -  2  1  -  -  -  - | -  -  -  -  1  1  - | -  1  1  -  -  -  - | -  -  -  -  1  1  - | -  -  2  2  -  -  - | -  -  1  -  -  1  - | -  1  1  -  -  -  - | -  -  1  -  -  -  1 |
| Gender  Male  Female | 4  2 | 4  - | 4  - | 1  1 | -  2 | 3  - | 2  - | 1  1 | -  2 | 1  1 | 1  1 | 3  - | 2  - | 1  3 | 2  1 | 2  1 | 16  5 | 9  5 | -  2 | 2  1 | -  2 | 1  2 | 2  2 | 1  1 | 1  2 | 2  - | -  2 | 2  - | 3  1 | 2  - | 1  1 | -  2 |
| Geographic region  Groningen, Friesland, Drenthe  Overijssel, Gelderland  Utrecht  North-Holland, Flevoland  South-Holland  South  South East | -  -  -  6  -  -  - | -  1  -  1  -  1  1 | 1  -  1  1  -  1  - | -  -  -  2  -  -  - | 1  -  -  -  -  -  1 | -  -  -  -  1  2  - | -  1  -  -  1  -  - | -  -  -  -  -  2  - | -  -  -  -  -  2  - | 1  1  -  -  -  -  - | 1  -  -  1  -  -  - | -  -  -  2  1  -  - | -  -  2  -  -  -  - | -  1  1  -  1  -  1 | -  2  -  -  -  -  1 | -  -  -  2  1  -  - | 1  8  -  4  5  2  1 | 1  2  1  4  1  4  1 | -  1  -  1  -  -  - | 1  -  -  2  -  -  - | 1  -  -  -  1  -  - | 1  -  1  -  1  -  - | -  1  -  2  -  1  - | -  -  1  -  -  -  1 | -  -  -  1  1  -  1 | -  -  -  2  -  -  - | -  -  -  2  -  -  - | -  -  -  -  2  -  - | -  -  -  3  -  1  - | -  -  -  1  -  1  - | -  1  -  1  -  -  - | -  -  -  1  1  -  - |
| Ethnicity  Dutch  First generation migrant  Second generation migrant | 2  4  - | -  4  - | -  4  - | -  2  - | -  1  1 | -  3  - | -  2  - | 1  1  - | -  2  - | -  2  - | -  2  - | -  1  2 | -  2  - | -  4  - | 3  -  - | 1  2  - | -  21  - | -  14  - | -  2  - | -  3  - | -  2  - | -  3  - | 1  2  - | -  2  - | -  2  1 | -  2  - | -  2  - | 1  1  - | -  4  - | 1  1  - | -  2  - | 1  1  - |
| Country of birth  Netherlands  Surinam  Eritrea/Ethiopia  Somalia  Indonesia  Morocco  Pakistan  Nepal  USA  Vietnam  Ukraine  Afghanistan  Former Yugoslavia  Gambia  Curacao  Romania  Myanmar  Sudan  Ghana  Libya  Italy  Liberia  Greece | 3  2  -  -  -  -  -  -  -  -  -  -  -  -  -  -  -  -  -  -  1  -  - | -  -  4  -  -  -  -  -  -  -  -  -  -  -  -  -  -  -  -  -  -  -  - | -  -  4-  -  -  -  -  -  -  -  -  -  -  -  -  -  -  -  -  -  -  - | -  -  -  -  -  1  -  -  -  -  -  -  -  -  -  -  -  -  -  -  -  1  - | 1  -  1  -  -  -  -  -  -  -  -  -  -  -  -  -  -  -  -  -  -  -  - | -  -  3  -  -  -  -  -  -  -  -  -  -  -  -  -  -  -  -  -  -  -  - | -  -  2  -  -  -  -  -  -  -  -  -  -  -  -  -  -  -  -  -  -  -  - | 1  -  -  -  -  -  -  -  -  -  -  -  -  -  -  -  -  -  -  -  -  -  1 | -  -  1  -  2  -  -  -  -  -  -  -  -  -  -  -  -  -  -  -  -  -  - | -  -  2  -  -  -  -  -  -  -  -  -  -  -  -  -  -  -  -  -  -  -  - | -  -  -  2  -  -  -  -  -  -  -  -  -  -  -  -  -  -  -  -  -  -  - | 2  -  -  -  1  -  -  -  -  -  -  -  -  -  -  -  -  -  -  -  -  -  - | -  -  -  -  -  2  -  -  -  -  -  -  -  -  -  -  -  -  -  -  -  -  - | -  -  4  -  -  -  -  -  -  -  -  -  -  -  -  -  -  -  -  -  -  -  - | 3  -  -  -  -  -  -  -  -  -  -  -  -  -  -  -  -  -  -  -  -  -  - | 1  -  -  -  -  -  1  1  -  -  -  -  -  -  -  -  -  -  -  -  -  -  - | -  -  18  1  -  -  -  -  -  -  -  -  -  -  -  -  -  1  -  -  -  -  - | -  -  5  9  -  -  -  -  -  -  -  -  -  -  -  -  -  -  -  -  -  -  - | -  -  -  -  -  -  -  -  1  1  -  -  -  -  -  -  -  -  -  -  -  -  - | -  -  2  1  -  -  -  -  -  -  -  -  -  -  -  -  -  -  -  -  -  -  - | -  -  2  -  -  -  -  -  -  -  -  -  -  -  -  -  -  -  -  -  -  -  - | -  1  -  -  -  -  -  -  -  -  1  1  -  -  -  -  -  -  -  -  -  -  - | 2  -  -  -  -  1  -  -  -  -  -  -  1  -  -  -  -  --  -  -  -  - | -  -  1  -  -  -  -  -  -  -  -  1  -  -  -  -  -  -  -  -  -  -  - | 1  -  -  -  -  1  -  -  -  -  -  -  -  1  -  -  -  -  -  -  -  -  - | -  2  -  -  -  -  -  -  -  -  -  -  -  -  -  -  -  -  -  -  -  -  - | -  -  -  -  -  -  2  -  -  -  -  -  -  -  -  -  -  -  -  -  -  -  - | 1  -  -  -  -  -  -  -  -  -  -  -  -  -  1  -  -  -  -  -  -  -  - | -  -  -  1  -  -  -  -  -  -  -  -  -  -  -  -  -  -  1  1  -  -  - | 1  -  -  -  -  -  -  -  -  -  -  -  -  -  -  1  -  -  -  -  -  -  - | -  -  2  -  -  -  -  -  -  -  -  -  -  -  -  -  -  -  -  -  -  -  - | 1  -  -  -  -  -  -  -  -  -  -  -  -  -  -  -  1  -  -  -  -  -  - |
| Risk group  Contact of tuberculosis patient  Immigrant  Asylum seeker  Undocumented migrant  Homeless  Alcohol addict  Drugs addict  Travel to endemic regions >3 mo  Prisoner | 2  -  -  -  1  -  -  -  - | -  -  4  -  -  -  -  -  - | -  -  4  -  -  -  -  -  - | -  -  -  -  -  -  -  -  - | 2  -  1  -  -  -  --  - | -  1  2  -  -  -  -  -  - | -  -  2  -  -  -  -  -  - | -  -  -  -  -  -  -  -  - | -  1  1  -  -  -  -  -  - | -  -  2  -  -  -  -  -  - | -  -  1  -  -  -  -  -  - | -  -  -  1  1  -  -  -  - | -  -  -  -  -  -  -  1  - | 1  -  2  -  -  -  -  -  - | 1  -  -  -  -  -  -  -  - | -  -  -  -  1  -  -  -  - | 5  -  18  -  -  -  -  -  - | 3  -  4  -  -  -  -  -  - | -  -  -  -  -  -  -  -  - | -  -  3  -  -  -  -  -  - | 1  -  2  -  -  -  -  -  - | -  -  1  -  1  -  -  -  1 | -  -  -  -  -  -  -  -  - | -  -  1  -  -  -  -  -  - | -  1  1  -  -  -  -  -  - | -  -  -  -  -  1  1  -  - | -  -  -  -  -  -  -  -  - | -  -  -  -  -  1  -  -  - | -  -  -  3  1  -  -  -  - | -  -  -  -  -  -  -  -  - | -  1  1  -  -  -  -  -  - | -  -  -  -  -  -  -  -  - |
| Diagnosis  PTB  ETB  PTB+ETB | 2  2  2 | 1  2  1 | 2  2  - | -  2  - | -  -  2 | 2  1  - | 1  1  - | 2  -  - | -  2  - | 1  1  - | 1  1  - | 1  1  1 | -  1  1 | 2  1  1 | -  1  2 | 2  1  - | 13  4  4 | 5  8  1 | -  2  - | -  2  1 | -  2  - | 3  -  - | 1  2  1 | 1  -  1 | 2  1  - | -  2  - | 1  -  1 | 1  -  1 | -  3  1 | 1  -  1 | -  2  - | 2  -  - |

PTB: pulmonary tuberculosis; ETB: extra-pulmonary tuberculosis

^1^This cluster contains two epidemiological linked cases from the same geographical area and one non-epidemiological linked case from a different geographical area.

^2^This cluster contains ten epidemiological linked cases from the same geographical area and eleven non-epidemiological linked cases of which four cases from a different geographical area.

^3^This cluster contains four epidemiological linked cases from the same geographical area and ten non-epidemiological linked cases from different geographical areas.

^4^This cluster contains three epidemiological linked cases from the same geographical area and one non-epidemiological linked case from a different geographical area.
